# Distinct *EGFR* Mutation Pattern in Patients With Non-Small Cell Lung Cancer in Xuanwei Region of China: A Systematic Review and Meta-Analysis

**DOI:** 10.3389/fonc.2020.519073

**Published:** 2020-11-02

**Authors:** Li Lv, Zhichao Liu, Yang Liu, Wenhui Zhang, Lifeng Jiang, Tingting Li, Xinyan Lu, Xuefen Lei, Wenhua Liang, Jie Lin

**Affiliations:** ^1^ Department of Medical Oncology, The Second Affiliated Hospital of Kunming Medical University, Kunming, China; ^2^ School of Medicine, Shanghai Jiao Tong University, Shanghai, China; ^3^ Department of Thoracic Surgery and Oncology, The First Affiliated Hospital of Guangzhou Medical University, Guangzhou, China; ^4^ Guangzhou Institute of Respiratory Disease and China State Key Laboratory of Respiratory Disease and National Clinical Research Center for Respiratory Disease, Guangzhou, China

**Keywords:** lung cancer, *EGFR* mutation, subtype, China, Xuanwei

## Abstract

**Background:**

In the Xuanwei region of China, lung cancer incidence and mortality are among the highest in China, attributed to severe air pollution generated by combustion of smoky coal. No study has yet comprehensively evaluated the prevalence of epidermal growth factor receptor (*EGFR*) mutation characteristics in patients with non-small cell lung cancer (NSCLC) in Xuanwei. This meta-analysis was designed to analyze the *EGFR* mutation pattern in NSCLC patients in Xuanwei region of Yunnan Province in China.

**Methods:**

Electronic databases were comprehensively searched and relevant literatures were retrieved. The odds ratio (OR) for *EGFR* mutations between Xuanwei region and non-Xuanwei region was calculated, and the absolute incidence of *EGFR* mutations in Xuanwei was pooled by mutation subtype.

**Results:**

Seven studies involving 1,355 patients with NSCLC from Yunnan Province (442 in Xuanwei and 913 in other regions) were included. The *EGFR* mutation rate ranged between 30.19% and 55.56%. Higher uncommon *EGFR* mutations (OR: 5.69, 95%CI: 2.23–14.49, P<0.001) and lower common *EGFR* mutations (OR: 0.18, 95%CI: 0.07–0.45, P<0.001) were found in Xuanwei region, compared with non-Xuanwei region. Specifically, the uncommon *EGFR* mutation rate was 59.50% and common *EGFR* mutation rate was 40.50% in Xuanwei. The mutation incidence of exon 18 G719X (OR: 3.21, 95%CI: 1.48–6.97, P=0.003), exon 20 S768I (OR: 6.44; 95%CI: 2.66–15.60; P<0.001), and exon 18 G719X + 20 S768I (OR: 6.55; 95%CI: 1.92–22.33; P=0.003) in Xuanwei were significantly higher, while the frequency of 19 deletion (OR: 0.28, 95%CI: 0.11–0.77, P<0.001) and 21 L858R mutation (OR: 0.51, 95%CI: 0.31–0.84, P=0.007) were lower.

**Conclusions:**

The results highlight the distinct *EGFR* mutation spectrum of NSCLC patients in Xuanwei region compared with other regions, with higher uncommon mutations but lower common mutations. The distinct Xuanwei featured genetic variations provide a unique model to further study carcinogenesis of lung cancer.

## Introduction

Lung cancer has the highest morbidity and is the leading cause of cancer-related death worldwide, with 85% of patients having non-small-cell lung cancer (NSCLC) (1, 2). With the advances in molecular oncology, multiple genetic variants have been determined as therapeutic targets for lung cancer, and many onco-targeted drugs had been developed. Epidermal growth factor receptor (EGFR) is a well-accepted carcinogenic variant and driver gene in lung cancer. NSCLC patients with activating *EGFR* mutations are identified in about 40~60% of Asian and 10% of Western populations (3). When EGFR is activated, it can trigger intracellular signaling cascades that affect cellular proliferation, angiogenesis, and apoptosis through transmembrane receptors (4). Studies showed that EGFR tyrosine kinase inhibitors (TKIs) confer better outcome in patients with the *EGFR* common mutations (exon 19 deletion, exon 21 L858R point mutation) (5). A genetic divergence of *EGFR* mutation rates was demonstrated according to ethnicity in previous research (6, 7), and the frequency of *EGFR* was highest among Asians (47%) and lowest among Oceanians (12%) (8).

Xuanwei is a small city located in Yunnan Province of China. This region is one of the major coal-producing regions in Yunnan Province and renowned for distinct lung cancer characteristics (9–11): first, lung cancer incidence in Xuanwei region is regionally specific with a high incidence rate and mortality rate. Second, the mortality rate of females is relatively high and almost all of them are never smokers, 20 times higher than other regions of China, and it is among the highest in the world for female lung cancer mortality. Additionally, the onset of lung cancer happens at a relatively younger age in the Xuanwei region, which occurs in younger than the peak age of onset of lung cancer in other parts of China by more than 10 years (12). Environmental factors are known to play a role in lung cancer development, and indoor air pollution from the use of smoky coal for household purposes has been suggested to be the cause of the high incidence of lung cancer in Xuanwei, especially in female patients with non-smoking history (13). Previously, a small-scale study revealed that non-smoking female NSCLC patients in Xuanwei harbored different *EGFR* mutation patterns when compared with other parts of Asia (14), suggesting that there may exist distinct genetic background in this ethnic group, certain susceptible populations, and unique environments. Therefore, it is of great significance to study the specific *EGFR* mutation patterns in NSCLC patients from the Xuanwei region of China, which may lead to better understanding the carcinogenesis of *EGFR*-mutated NSCLC and more effective targeted therapeutic interventions.

The *EGFR* mutation of NSCLC patients in Xuanwei region has not been fully understood; thus, we conducted this meta-analysis and systematic review to probe further into the *EGFR* mutation pattern of NSCLC patients in the Xuanwei region compared with the non-Xuanwei population in Yunnan Province to further effectively guide clinical treatment.

## Methods

### Study design

To obtain a more precise estimate of EGFR mutation pattern in NSCLC patients of Xuanwei region, we pooled the prevalence of *EGFR* mutation (common and uncommon type) in Xuanwei region and control region (non-Xuanwei) in the rest of Yunnan Province where the level of air pollution and lung cancer incidence and mortality were comparable to most parts of China.

Common EGFR mutations (or classic mutations) are deemed as exon 19 deletion and exon 21 L858R substitution, accounting for approximately 90% of EGFR mutations in NSCLC (15, 16). Uncommon EGFR mutations are deemed as mutations other than 19 deletion and 21 L858R, and they account for 10% to 20% of all EGFR mutations; the substitution mutations of G719X in exon 18, L861Q in exon 21, S768I in exon 20, exon 20 insertions, and complex mutations are the most frequent among the uncommon mutations (17–19).

### Search Strategy

A comprehensive literature search and systematic review of online databases PubMed, Embase, Web of Science, Medline, Cochrane Library, and Chinese Biomedical Literature Database was performed to identify relevant studies published before 6 November 2019 that examined EGFR mutation frequency in non-small cell lung cancer in southwest China’s Yunnan Province. The search key words including “EGFR” AND “mutation” AND (“NSCLC” OR “lung cancer”) AND (“Yunnan” OR “Xuanwei”) were used. No search limitations were set. The relevant abstracts and presentations from conferences were also manually searched. Then, we examined the publication of the list of references and searched for additional research.

### Inclusion and Exclusion Criteria

Eligible studies should meet the following criteria: (i) Publications describing the mutation subtype in *EGFR*-mutated NSCLC of Yunnan in southwestern China were retained. (ii) Mutation detection in paraffin-embedded tumor tissues or cytological specimens or blood samples can be included.

Studies were excluded if (i) data were insufficient to calculate the pooled incidence for this meta-analysis or (ii) they were review articles, case reports, editorials, expert opinions, non-comparative studies, unrelated to research topics, or duplicate reports.

### Data Extraction and Quality Assessment

This meta-analysis was conducted according to the Cochrane and Preferred Reporting Items for Systematic Reviews and Meta-Analyses guidelines (20). Two researchers (Li Lv and Zhichao Liu) independently performed all of the screening of studies and data extraction. The third researcher (Yang Liu) resolved the disagreements. For eligible research, we extract all available information: the first name of the author, year of publication, region, research type, number of patients, gender, age, smoking status, *EGFR* mutations, detection methods, tumor histology, disease stage, and metastases type. The quality of the included studies was assessed using the Agency for Healthcare Research and Quality Tool. Any disagreements were resolved through discussion and consensus.

### Statistical Analysis

Pooled odds ratio (OR) of common and uncommon *EGFR* mutation rate between Xuanwei region and non-Xuanwei region in Yunnan province of China was calculated, and the pooled frequency of *EGFR* mutations in Xuanwei was also calculated. Subgroup pooled OR and incidence were generated according to *EGFR* mutation subtypes. Cochran’s Q test and *I^2^* were used to estimate the heterogeneity effect among the studies. *I^2^* statistics more than 50% was suggestive of statistical heterogeneity between studies, and random effects model was used if heterogeneity existed. All tests were two-sided and a P value less than 0.05 was considered statistically significant. All statistical analyses were conducted with STATA 12.0 software (Stata Corporation, College Station, TX, USA) and R 3.4.1 software (R foundation for Statistical Computing, Vienna, Austria).

## Results

### The Selection and Characteristics of Study

**Figure 1:**
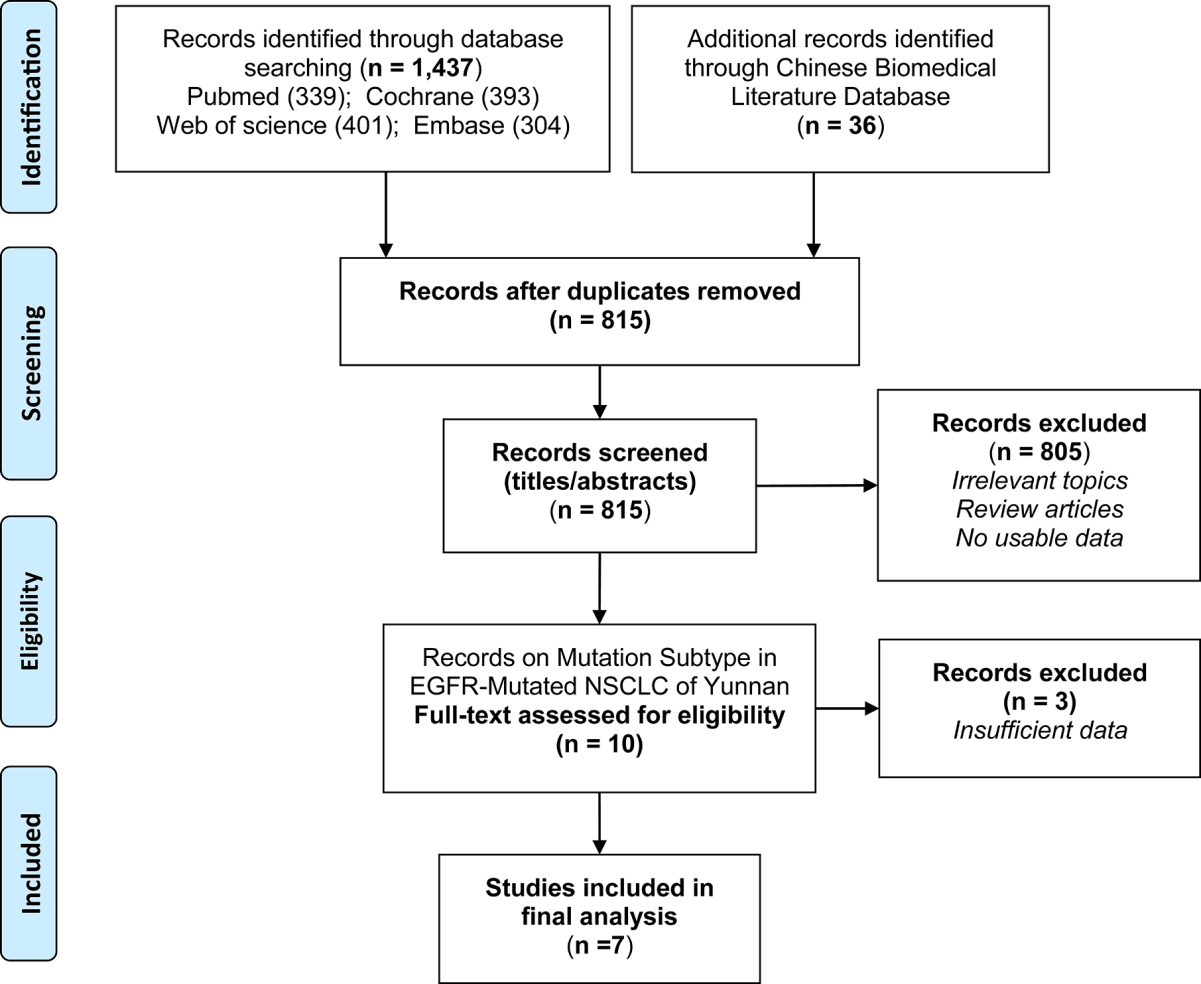
Flow chart detailing the search strategy and identification of studies.

A total of 1,473 publications were retrieved through the initial literature search, of which 658 papers were excluded due to duplication. With title and abstract review, 10 potentially relevant articles were identified for detailed review (13, 14, 21–28). These articles were further assessed for eligibility by reviewing the full texts, and 3 articles (23–25) were removed due to insufficient data. Finally, a total of 7 articles (13, 14, 21, 22, 26–28) were identified as eligible to be included in the meta-analysis (**Figure 1**).

**Table 1** summarized the characteristics of all included studies. Seven studies involving 1,355 NSCLC patients from different regions of Yunnan Province in southwestern China (442 from Xuanwei region, 913 from other region in the rest of Yunnan Province) were included. The majority of patients were adenocarcinoma and non-smoker. Four studies provided a comparison of *EGFR* mutations between Xuanwei and non-Xuanwei regions, while the other 3 studies (14, 26, 27) only evaluated *EGFR* mutations in Xuanwei region. In all included studies (13, 14, 21, 22, 26–28), the *EGFR* mutation status was mainly (5 out of 7) detected by Amplification Refractory Mutation System (ARMS), while next generation sequencing (NGS) or SNaPshot were used in the other 2 studies. Overall, the *EGFR* mutation positive rate was 30.19% to 55.56% across studies. All studies gained 10 to 11 scores in study quality assessment on a scale of 0 to 11 with the Agency for Healthcare Research and Quality Tool.

**Table 1:** Characteristics of the included studies.

First author	Publish year	Region	Study type	Number of patients			*EGFR* mutation			Female (%)	Age(years)	Smoker(%)	Adenocarcinoma (%)	Stage	Lymph node metastasis (%)	Brain metastasis (%)	*EGFR* mutation detection methods	Scores Of AHRQ
				Xuanwei	Non-Xuanwei	Total	Yes	No	Rate									
Guo et al. (27)	2019	China, Xuanwei	Retro	85	NA	85	39	46	45.88%	41.18%	54 (36-78)	NG	80.00%	NG	NG	NG	Targeted NGS	10
Zhou et al. (28)	2018	China, Yunnan	Retro	20	192	212	64	148	30.19%	38.21%	57.1 (31-86)	44.81%	81.60%	I, II, III, IV	NG	18.87%	Super ARMS-PCR and dd PCR	11
Zhou et al. (21)	2017	China, Yunnan	Retro	63	384	447	156	291	34.90%	44.97%	<65 (69.4%); 65-75 (23.5%); >75 (7.2%)	47.43%	86.58%	I, II, III, IV	NG	14.32%	ARMS-PCR	11
Chen et al. (13)	2016	China, Yunnan	Retro	90	168	258	124	134	48.06%	52.33%	<=60 (68.6%); >60 (31.4%)	27.13%	87.60%	I, II, III, IV	29.07%	NG	ARMS-PCR	10
Yang et al. (22)	2016	China, Yunnan	Retro	81	169	250	131	119	52.40%	NG	NG	NG	NG	NG	NG	NG	ARMS-Taqman	10
Yang et al. (26)	2016	China, Xuanwei	Retro	63	NA	63	35	28	55.56%	42.86%	<50 (54.0%); >=50 (46.0%)	39.68%	84.13%	NG	17.46%	NG	ARMS-Taqman	10
Hosgood et al. (14)	2013	China, Xuanwei	Retro	40	NA	40	14	26	35.00%	NG	46.5 ± 10	0	80.00%	NG	NG	NG	SNaPshot and separate PCR-based sizing technique	10

Retro, Retrospective study; EGFR, Epidermal growth factor receptor; NA, Not Available; NG, Not given; AHRQ, Agency for Healthcare Research and Quality; NGS, Next generation sequencing; ARMS, super amplification refractory mutation system; PCR, polymerase chain reaction; dd PCR, Droplet Digital polymerase chain reaction; GS, gene sequencing.

### Frequency and Odds Ratio of EGFR Mutation in Xuanwei and Non-Xuanwei Regions

**Figure 2:**
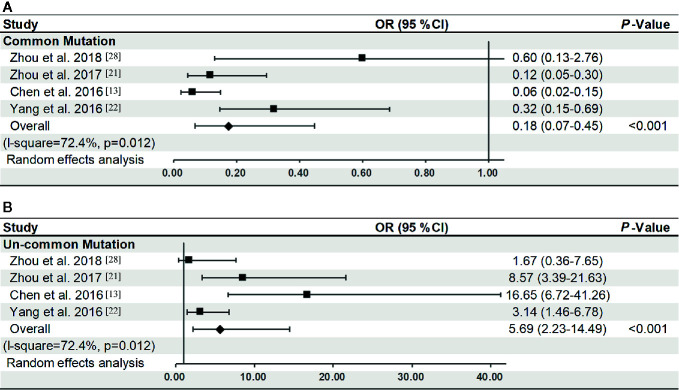
Odds of common **(A)** or uncommon **(B)** EGFR mutation of Xuanwei compared to non-Xuanwei regions (four studies). The center of each square is the odds ratio (OR) for individual trials and corresponding horizontal line is the 95% CI. The broken line and center of the blue diamond is overall pooled OR and the horizontal tip of the diamond is the 95% confidence interval (CI).

Four studies (13, 21, 22, 28) that simultaneously reported the *EGFR* mutation rate in Xuanwei and non-Xuanwei region were included in the comparative analysis. The difference in *EGFR* mutation rates is summarized according to regions (**Table 2**). One hundred and twenty-nine patients from Xuanwei region harbored *EGFR* mutations, with 64.34% (83/129) uncommon mutation and 35.66% (46/129) common mutation. In non-Xuanwei region, the incidence of uncommon and common *EGFR* mutation was 25.43% (88/346) and 74.57% (258/346) respectively. The frequency of uncommon mutations was higher in Xuanwei region than that in non-Xuanwei region (4 studies (13, 21, 22, 28) OR: 5.69, 95%CI: 2.23–14.49, P<0.001) (**Figure 2B**). By contrast, patients in Xuanwei regions were less likely to have common *EGFR* mutations (19 deletion or 21 L858R) compared with non-Xuanwei region (OR: 0.18, 95%CI: 0.07–0.45, P<0.001) (**Figure 2A**).

**Table 2:** Comparison of the incidence of *EGFR* mutation of NSCLC patients between Xuanwei and non-Xuanwei regions.

			Xuanwei				Non-Xuanwei				META		
			Yes	No	Total	Rate	Yes	No	Total	Rate	OR	95%CI	*P*-value
**Common mutation**	Zhou et al. (28)	2018	3	5	8	37.50%	28	28	56	50.00%	0.600	(0.131-2.755)	
	Zhou et al. (21)	2017	8	19	27	29.63%	101	28	129	78.29%	0.117	(0.046-0.295)	
	Chen et al. (13)	2016	14	37	51	27.45%	63	10	73	86.30%	0.060	(0.024-0.149)	
	Yang et al. (22)	2016	21	22	43	48.84%	66	22	88	75.00%	0.318	(0.148-0.686)	
	**Overall**		**46**	**83**	**129**		**258**	**88**	**346**		**0.176**	**(0.069-0.448)**	**<0.001**
											(**Heterogeneity**: *I^2 =^*72.4%, *p*=0.0012)		
													
**Uncommon mutation**	Zhou et al. (28)	2018	5	3	8	62.50%	28	28	56	50.00%	1.667	(0.363-7.652)	
	Zhou et al. (21)	2017	19	8	27	70.37%	28	101	129	21.71%	8.567	(3.393-21.628)	
	Chen et al. (13)	2016	37	14	51	72.55%	10	63	73	13.70%	16.650	(6.720-41.256)	
	Yang et al. (22)	2016	22	21	43	51.16%	22	66	88	25.00%	3.143	(1.458-6.777)	
	**Overall**		**83**	**46**	**129**		**88**	**258**	**346**		**5.685**	**(2.231-14.486)**	**<0.001**
											(**Heterogeneity**: *I^2 =^*72.4%, *p*=0.0012)		

OR, odds ratio; CI, confidence interval.

### Overall Incidence of Common and Uncommon EGFR Mutation in Xuanwei Region

To further illustrate the distribution of common and uncommon *EGFR* mutations in Xuanwei region, 7 studies (13, 14, 21, 22, 26–28) were included to calculate the pooled incidence (**Table 3**). A total of 217 patients had *EGFR* mutation in Xuanwei region. The pooled incidence of uncommon *EGFR* mutation was 59.5% (95%CI: 53.2%–65.9%), while the pooled incidence of common *EGFR* mutation was 40.5% (95%CI: 34.1%–46.8%).

**Table 3:** Incidence of common and uncommon *EGFR* mutation in Xuanwei region.

			Xuanwei				META		
			Yes	No	Total	Rate	Rate	95%CI
**Common mutation**	Guo et al. (27)	2019	22	17	39	56.41%	0.564	(0.408-0.720)
	Zhou et al. (28)	2018	3	5	8	37.50%	0.375	(0.040-0.710)
	Zhou et al. (21)	2017	8	19	27	29.63%	0.296	(0.124-0.469)
	Chen et al. (13)	2016	14	37	51	27.45%	0.275	(0.152-0.397)
	Yang et al. (22)	2016	21	22	43	48.84%	0.488	(0.339-0.638)
	Yang et al. (26)	2016	16	19	35	45.71%	0.457	(0.292-0.622)
	Hosgood et al. (14)	2013	6	8	14	42.86%	0.429	(0.169-0.688)
	**Overall**		**90**	**127**	**217**		**0.405**	**(0.341**-**0.468)**
							(**Heterogeneity**: *I^2 =^*48.0%, *p*=0.073)		
**Uncommon mutation**	Guo et al. (27)	2019	17	22	39	43.59%	0.436	(0.280-0.592)
	Zhou et al. (28)	2018	5	3	8	62.50%	0.625	(0.290-0.960)
	Zhou et al. (21)	2017	19	8	27	70.37%	0.704	(0.531-0.876)
	Chen et al. (13)	2016	37	14	51	72.55%	0.725	(0.603-0.848)
	Yang et al. (22)	2016	22	21	43	51.16%	0.512	(0.362-0.661)
	Yang et al. (26)	2016	19	16	35	54.29%	0.543	(0.378-0.708)
	Hosgood et al. (14)	2013	8	6	14	57.14%	0.571	(0.312-0.831)
	**Overall**		**127**	**90**	**217**		**0.595**	**(0.532-0.659)**
							(**Heterogeneity**: *I^2 =^*48.0%, *p*=0.073)		

### Subgroup Analysis of Different EGFR Mutation Subtypes in Xuanwei and Non-Xuanwei Regions

Patients from Xuanwei and non-Xuanwei regions with specific *EGFR* mutation subtypes were further analyzed (**Table 4**). Results showed that mutation of 18 G719X, 20 S768I, and 18 G719X + 20 S768I were more likely to appear in Xuanwei patients compared with non-Xuanwei patients (OR: 3.21, 95%CI: 1.48–6.97, P=0.003; OR: 6.44, 95%CI: 2.66–15.60, P<0.001, OR: 6.55, 95%CI: 1.92–22.33, P<0.05, respectively). In contrast, NSCLC patients of Xuanwei regions harbored lower frequency of 19 deletion (OR: 0.28, 95%CI: 0.11–0.77, P<0.001) and 21 L858R mutation (OR: 0.51, 95%CI: 0.31–0.84, P=0.007).

**Table 4:** Subgroup analysis of different *EGFR* mutation subtypes between Xuanwei and non-Xuanwei regions.

Mutation type		Guo et al. (27)	Zhou et al. (28)		Zhou et al. (21)		Chen et al. (13)		Yang et al. (22)		Yang et al. (26)	Hosgood et al. (14)	META(Xuanwei vs. Non-Xuanwei)			META(Xuanwei)	
		Xuanwei	Xuanwei	non-Xunwei	Xuanwei	non-Xunwei	Xuanwei	non-Xunwei	Xuanwei	non-Xunwei	Xuanwei	Xuanwei	OR^a^	95% CI	*P*-value	Rate^b^	95% CI
**Common**	19 deletion	56.41%	37.50%	26.79%	18.50%	45.00%	7.80%	49.30%	13.95%	39.77%	14.30%	28.57%	0.284	(0.105-0.766)	<0.001	0.13	(0.081-0.178)
	21 L858R		0	23.21%	11.10%	33.30%	19.60%	37.00%	34.88%	35.23%	31.40%	14.29%	0.511	(0.313-0.836)	0.007	0.197	(0.109-0.302)
**Uncommon**	18 G719X	7.69%	12.50%	1.79%	22.20%	3.90%	7.80%	1.40%	9.30%	7.95%	14.30%	50.00%	3.212	(1.481-6.965)	0.003	0.11	(0.070-0.151)
	20 T790M	0	12.50%	14.29%	0	2.30%	0	0	4.65%	4.55%	2.90%	0	0.927	(0.286-2.999)	0.899	0.0076	(0-0.034)
	20 S768I	0	25.00%	3.57%	11.10%	3.10%	3.90%	2.70%	23.26%	1.14%	17.10%	0	6.437	(2.657-15.598)	<0.001	0.109	(0.036-0.207)
	20 insertion	0	0	1.79%	0	3.90%	2.00%	0	0	0	0	7.14%	1.335	(0.316-5.634)	0.694	0.0014	(0-0.021)
	21 L816Q	0	0	0	3.70%	2.30%	2.00%	2.70%	0	0	0	0	1.395	(0.339-5.741)	0.644	0.0022	(0-0.023)
	18 G719X + 20 S768I	15.38%	12.50%	10.71%	18.40%	1.60%	45.10%	4.10%	9.30%	3.41%	17.10%	0	6.549	(1.921-22.332)	0.003	0.213	(0.048-0.439)
	18 G719X + 20 G779C	5.13%	0	0	0	0	2.00%	0	0	0	0	0	3.969	(0.603-26.132)	0.152	0.0035	(0-0.023)
	18 G719X + 21 L816Q	2.56%	0	0	0	0.80%	2.00%	1.40%	0	0	0	0	1.959	(0.366-10.489)	0.432	0.0017	(0-0.019)
	18 G719X + 21 L858R	0	0	0	3.70%	0	0	0	0	0	0	0	4.421	(0.736-26.554)	0.104	0	(0-0.013)
	18 G719X + 21 L858R + S768I	0	0	0	3.70%	0	0	0	0	0	0	0	4.421	(0.736-26.554)	0.104	0	(0-0.013)
	19 deletion + 21 L858R	0	0	1.79%	0	0.80%	2.00%	0	0	4.55%	0	0	0.95	(0.242-3.731)	0.941	0.0001	(0-0.016)
	19 deletion + 20 T790M	0	0	10.71%	0	2.30%	0	0	0	0	0	0	0.776	(0.156-3.852)	0.756	0	0
	20 S768I + 20 T790M	0	0	0	7.40%	0.00%	0	0	0	0	0	0	6.046	(1.119-32.677)	0.037	0.0002	(0-0.017)
	20 S768I +20 insertion	0	0	0	0	0	3.90%	1.40%	0	0	0	0	3.284	(0.607-17.760)	0.167	0.0013	(0-0.021)
	20 S768I + 21 L858R	0	0	0	0	0.80%	2.00%	0	0	0	0	0	2.963	(0.534-16.429)	0.214	0.0001	(0-0.016)
	20 T790M + 21 L858R	0	0	5.36%	0	0	0	0	4.65%	3.41%	2.90%	0	1.431	(0.374-5.472)	0.601	0.0048	(0-0.029)

OR, odds ratio; CI, confidence interval.

a the pooled OR of EGFR mutation for Xuanwei region vs. non-Xuanwei region.

b the pooled incidence of EGFR mutation in Xuanwei region.

## Discussion

Due to the low incidence of NSCLC with the so-called uncommon *EGFR* mutations in the general population, information on their significance of carcinogenesis and treatment is still incomplete and deserves further investigation. We conducted a systematic review and meta-analysis of current researches (13, 14, 21, 22, 26–28) to evaluate the mutation pattern of *EGFR*-mutated NSCLC of Yunnan province in southwestern China. The pooled analysis confirmed a distinct *EGFR* mutation spectrum in Xuanwei region. The NSCLC patients in Xuanwei region are present with significantly higher incidence of uncommon *EGFR* mutations, especially 18 G719X mutation, 20 S768I mutation, and their combined mutation, but lower incidence of the two common mutations (19 deletion and 21 L858R substitution), providing a unique model for uncommon-*EGFR*-mutation-related lung cancer.

*EGFR* mutations in NSCLC is one of the most common genetic variations, especially in East Asians, females, and non-smokers (29). Studies suggested that NSCLC patients with *EGFR* mutation were significantly related to adenocarcinoma and light smoking, rather than gender (30). It was suggested that the dominant mutation rate of *EGFR* in women is a reflection of a higher frequency of adenocarcinoma (31). The results of our analysis revealed that the overall *EGFR* mutation rate of NSCLC patients varied from 30.2% to 55.6% in Yunnan province (Xuanwei region: 40.0%–56.7%; non-Xuanwei region: 29.2%–52.1%), which was in the range of other reports in East Asian areas (31%–56%) (8, 31–34) and similar to other studies performed in other regions of China (ranging from 33.64% to 53.69%)(**Table S1**). Most patients covered by our survey were carriers of adenocarcinomas (ranging from 80.0% to 87.6%) and female (ranging from 38.2% to 52.3%), which was similar to other studies performed in East Asian countries (8, 33, 34). Notably, despite similar incidence of overall *EGFR* mutations, the NSCLC patients in Xuanwei region present a distinctive characteristic with higher uncommon *EGFR* mutations and lower common *EGFR* mutations (**Table 2**, **Table S2**).

Generally, exon 19 deletions and 21 L858R substitutions, accounting for approximately 90% of *EGFR* mutations in NSCLC, are termed common or classic mutations and lead to high sensitivity to *EGFR* TKIs (35). Other *EGFR* mutations are termed uncommon mutations, accounting for 10%–20% of all *EGFR* mutations, and patients with uncommon *EGFR* mutation are a heterogeneous group exhibiting different responses to *EGFR* TKIs (36, 37). There are significant regional differences in *EGFR* gene mutation in China. It has been reported that exon 21 of *EGFR* gene mutation is dominant in Taiwan (38), while exon 19 is dominant in Yunnan (21) and exon 20 is dominant in Guangdong (36). Our results show that *EGFR* gene mutation mainly occurred in exon 21 L858R mutation and in exon 19 deletion NSCLC patients in most regions of Yunnan, which was consistent with other reports. Another study showed that the uncommon *EGFR* mutations were present in 11.9% of all patients with documented *EGFR* mutation (34%) in a Chinese NSCLC cohort (36). However, according to our study, the detection rate of uncommon EGFR mutations varied from 43.6% to 72.6% in NSCLC patients in Xuanwei, which was significantly higher than that of the general Chinese population and other regions of the world (ranging from 3.4% to 15.5%) (**Table 2**, **Table S2**). This result further emphasizes the uniqueness of the Xuanwei population in lung cancer tumorigenesis.

The majority of clinical trials evaluating the efficacy of *EGFR* TKIs have included only patients with common *EGFR* mutations due to the low incidence of uncommon *EGFR* mutations in the general population; thus, the efficacy of *EGFR* TKIs in patients with uncommon *EGFR* mutations remains elusive. A post-hoc study showed that NSCLC patients with uncommon *EGFR* mutation (L861Q or G719X) was significantly associated with shorter overall survival (11.9 vs. 29.3 months) compared with patients harboring common *EGFR* mutation (L858R or 19 deletion) in the EGFR TKI treatment group (39). The patients with uncommon *EGFR* mutation (41% vs. 62%–83%) had a significantly lower overall response rate (ORR) of *EGFR* TKIs and shorter progression-free survival (PFS) (2.2 vs. 11.4 months) than that in those with common *EGFR* mutation (39). Additionally, in other studies, a poor ORR and shorter PFS were also found in uncommon *EGFR* mutations compared with the classic *EGFR* mutations. However, a similarly poor response to *EGFR* TKIs was observed in other uncommon mutation rather than G719X and L861Q (31, 40). It has also been documented that patients with NSCLC *EGFR* mutation in Xuanwei have poor prognosis after treatment with *EGFR* TKIs (21), possibly owing to the high incidence of uncommon *EGFR* mutations in this area.

Recent studies have suggested that the lung cancer in Xuanwei region showed distinct mutational signatures and signaling pathways, and two single nucleotide variants located in exon 20 and exon 18 of the *EGFR* gene were known to be related to lung adenocarcinoma in Xuanwei (41). In line with this, our results also suggested that patients with exon 20 S768I and exon 18 G719X mutations presented higher mutation rates than those from other regions. The specific genotypes of complex *EGFR* mutations (double or multiple concomitant *EGFR* mutations) are diverse, which can be common single mutation combined with common single mutation, common single mutation combined with rare single mutation, rare single mutation combined with rare single mutation, or compound mutation combined with known resistance genes. In this study, in patients with complex mutations, there was only one case with the common mutation combination (19deletion + L858R), while the others were the rare mutation combination, mainly including G719X, S768I, T790M, 20 insertions, and L861Q. Specifically, the 20 S768I mutation, 18 G719X mutation, co-mutation in 20 S768I + 18 G719X, and co-mutation in 20 S768I + 20 T790M were significantly more likely to present in Xuanwei patients compared with non-Xuanwei patients (all *P<*0.05). These mutations were thought to promote the development and progression of lung cancer (42). For other uncommon *EGFR* mutation types, there was no statistically significant difference between Xuanwei and non-Xuanwei regions. However, it should be noted that some co-mutations with uncommon *EGFR* mutation did present an un-negligible high OR value in Xuanwei region, such as 18 G719X+21 L858R (OR: 4.42), 18 G719X + 20 G779C (OR: 3.97), and 20 S768I +20 insertion (OR: 3.28). In other ways, the 20 S768I + 20 T790M was significantly more frequently present in Xuanwei region (OR, 6.05) whereas 19 deletion + 20 T790M tends to be present less in Xuanwei region (OR, 0.77), which may reflect an acquired resistance after *EGFR*-TKI therapy in these NSCLC patients with prevalent uncommon *EGFR* mutation in Xuanwei region. Overall, these results revealed a relatively high mutation frequency of so-called uncommon mutations (e.g., 18 G719X, 20 S768I, or 18 G719X/20 S768I in conjunction with other mutation) in the unique Xuanwei population, strikingly divergent from those in other populations from Asia. Given that our subjects (Xuanwei population) live in an area where coal is typically burned indoors, our analysis implies that the tumorigenesis and progression of lung cancer in Xuanwei region is different from that in other geographic areas, which may due to its distinctive etiology and the different environmental exposures.

The main difference between environmental exposure may be related to indoor solid fuel use related to indoor air pollution. In Xuanwei, indoor air pollution from bituminous coal burning in unvented fire pits was suggested to be the main cause of high lung cancer mortality (11). Polycyclic aromatic hydrocarbons (PAHs)-DNA adducts have been observed in the bronchoalveolar lavage fluid of coal-burning residents in Xuanwei region (11). Previous study showed that the mutations in *EGFR* exons 21 and 18 were associated with emissions from coal combustion (14). In addition, PAHs have been found to increase intracellular calcium in human cells (43), which may result in EGFR-dependent cell proliferation (4), suggesting that PAHs may lead to a unique mutation pattern. Liu et al. (44) evaluated the relationship among indoor air pollution, tobacco use, and lung cancer risk, showing that the risk association between smoking and lung cancer increased with the decrease of bituminous coal consumption. In other words, the relationship between smoking and lung cancer is relatively weak when there is a strong correlation between lung cancer and bituminous coal. Xuanwei is located in one of China’s largest tobacco producing provinces (45). In Xuanwei, the smoking rates of males in high, medium, and low incidence areas were 75%, 78%, and 63% respectively, and the exposure rates of second-hand smoke were 85%, 88%, and 58%, which were much higher than the national average level of male smoking rate (52.1%) and second-hand smoke exposure level (72.4%) (46). The discrepancy of mutation subtypes may provide clues for the mechanism of the occurrence of EGFR mutation. And this comparative analysis provides reference data, allowing a better understanding of a possible mechanism of *EGFR* mutation in Xuanwei, paving the way toward better exploration of uncommon EGFR mutation in lung cancer pathogenesis.

There are several limitations to the study. First, all included publications were retrospective studies with inherent biases. Second, the sample size of the study was not big enough. Third, only one study used NGS method to detect EGFR mutation; most of the included studies applied ARMS method to detect EGFR mutation, which is limited to the detection of known mutations and therefore might miss some rare EGFR mutations. Nevertheless, it should be noted that the detection methods in this analysis were already sufficient for the detection of classic (19 deletions and 21 L858R) and those known non-classic *EGFR* mutations in exons 18 to 21. A high concordance was found between ARMS and NGS, but more detailed information (unknown genetic mutations and deep sequencing) could be revealed by NGS (30, 47, 48). Therefore, a larger amount of sample and adoption of NGS is needed to validate the results of this study.

In conclusion, our analysis suggested the prevalence of *EGFR* mutation in Xuanwei region that is differentiating it from the general population. The frequency of S768I, G719X, and G719X+S768I were higher, but the 19 deletions and L858R mutations were lower in Xuanwei region. The difference of EGFR mutation between patients in Xuanwei region and in other areas may indicate the difference of lung cancer pathogenesis, dietary habits, coal-burning factors, or genetic backgrounds, which are worthy of more detailed studies.

## Data Availability Statement

The raw data supporting the conclusions of this article will be made available by the authors, without undue reservation, to any qualified researcher.

## Author Contributions

JL and WL designed and financed the study. LL performed the literature search and review, data extraction, and drafted the manuscript. ZL performed all of the screening of studies and data extraction. Any disagreements were resolved by YL. WZ, LJ, and TL were responsible for the analysis of pooled data. All authors contributed to the article and approved the submitted version.

## Funding

This study was supported by the Research of Yunnan Provence Science and Technology Planning Project (Project No.2017FE468 (-201), Project No.202001AT070027); the National Natural Science Foundation of China (Project No. 81960423) and the Second Affiliated Hospital of Kunming Medical University Program (grant no. 2019YK001).

## Conflict of Interest

The authors declare that the research was conducted in the absence of any commercial or financial relationships that could be construed as a potential conflict of interest.
