# Supplementary material for: Distinct EGFR Mutation Pattern in Patients With Non-Small Cell Lung Cancer in Xuanwei Region of China: A Systematic Review and Meta-Analysis
Source: Front Oncol. 2020 Nov 2;10:519073. doi: 10.3389/fonc.2020.519073 (PMC7667261; doi:10.3389/fonc.2020.519073)
Supplement: Supplementary file 1 [file Table_1.docx]

**Table S1. The incidence of EGFR mutation of NSCLC patients** **in China and other major regions worldwide.**

| **First author** | **Publish year** | **Region** | **Study type** | **Number of patients** | **EGFR mutation** | | |
| --- | --- | --- | --- | --- | --- | --- | --- |
|  |  |  |  |  | **Yes** | **No** | **Rate** |
|  |  |  |  |  |  |  |  |
| Kate et al.[1] | 2019 | India | Retro | 5738 | 1260 | 4478 | 21.96% |
| Chantharasamee et al.[2] | 2019 | Thailand | Retro | 681 | 317 | 364 | 46.55% |
| Suh et al.[3] | 2018 | USA | Retro | 6832 | 1342 | 5490 | 19.64% |
| Naderi et al.[4] | 2015 | Lebanon | Retro | 201 | 24 | 177 | 11.94% |
| Lai et al.[5] | 2013 | China | Retro | 697 | 235 | 462 | 33.72% |
| Zhang et al.[6] | 2018 | Beijing, China | Retro | 110 | 37 | 73 | 33.64% |
| Wu et al.[7] | 2016 | Taiwan, China | Retro | 3146 | 1689 | 1457 | 53.69% |
| Tu et al.[8] | 2017 | Guangzhou, China | Retro | 5363 | 1837 | 3526 | 34.25% |
| Wei et al.[9] | 2017 | Henan, China | Retro | 456 | 200 | 256 | 43.90% |

NA, Not Available

| **Table S2. Incidence of common and uncommon EGFR mutation in China and other major regions worldwide.** | | | | | | | | |
| --- | --- | --- | --- | --- | --- | --- | --- | --- |
| **EGFR mutation** | **First author** | **Publish year** | **Region** | **Rate** | **Female (%)** | **Age(years)** | **Smoker(%)** | **Adenocarcinoma (%)** |
| **Common mutation** | Kate et al.[1] | 2019 | India | 93.41% | NA | NA | NA | NA |
|  | Chantharasamee et al.[2] | 2019 | Thailand | 91.17% | NA | NA | NA | NA |
|  | Naderi et al.[4] | 2015 | Lebanon | 91.67% | NA | NA | NA | NA |
|  | Kobayashi et al.[10] | 2015 | Japan | 87.23% | NA | NA | NA | NA |
|  | John et al.[11] | 2018 | 18 countries | 96.59% | NA | NA | NA | NA |
|  | Lai et al.[5] | 2013 | China | 91.49% | 53.95% | NA | 26.05% | 66.05% |
|  | Wu et al.[7] | 2016 | Taiwan, China | 86.38% | NA | NA | NA | NA |
|  | Tu et al.[8] | 2017 | Guangzhou, China | 88.13% | 55.60% | 59(23-91) | 24.30% | 95.20% |
|  | Wei et al.[9] | 2017 | Henan, China | 84.50% | 66.27% | NA | 23.08% | 98.22% |
|  |  |  |  |  |  |  |  |  |
|  |  |  |  |  |  |  |  |  |
| **Uncommon mutation** | Kate et al.[1] | 2019 | India | 6.59% | 41.00% | 55(25-82) | 20.50% | 96.40% |
|  | Chantharasamee et al.[2] | 2019 | Thailand | 8.83% | 35.70% | 68(53-80) | 50.00% | 96.40% |
|  | Naderi et al.[4] | 2015 | Lebanon | 8.33% | NA | NA | NA | NA |
|  | Kobayashi et al.[10] | 2015 | Japan | 12.77% | NA | NA | NA | NA |
|  | John et al.[11] | 2018 | 18 countries | 3.41% | NA | NA | NA | NA |
|  | Lai et al.[5] | 2013 | China | 9.30% | 55.00% | NA | 40.00% | 65.00% |
|  | Wu et al.[7] | 2016 | Taiwan | 13.62% | NA | NA | NA | NA |
|  | Tu et al.[8] | 2017 | Guangzhou, China | 11.87% | 45.90% | 59(31-86) | 30.70% | 91.80% |
|  | Wei et al.[9] | 2017 | Henan, China | 15.50% | 54.84% | NA | 29.03% | 100.00% |

NA, Not Available

**References**

1. Kate S, Chougule A, Joshi A, et al. Outcome of uncommon EGFR mutation positive newly diagnosed advanced non-small cell lung cancer patients: a single center retrospective analysis. Lung Cancer (Auckl). 2019 Jan 29;10:1-10. doi: 10.2147/LCTT.S181406.
2. Chantharasamee J, Poungvarin N, Danchaivijitr P, et al. Clinical outcome of treatment of metastatic non-small cell lung cancer in patients harboring uncommon EGFR mutation. BMC Cancer. 2019 Jul 17;19(1):701. doi: 10.1186/s12885-019-5913-9.
3. Suh JH, Schrock AB, Johnson A, et al. Hybrid Capture-Based Comprehensive Genomic Profiling Identifies Lung Cancer Patients with Well-Characterized Sensitizing Epidermal Growth Factor Receptor Point Mutations That Were Not Detected by Standard of Care Testing. Oncologist. 2018 Jul;23(7):776-781. doi: 10.1634/theoncologist.2017-0493. Epub 2018 Mar 14.
4. Naderi S, Ghorra C, Haddad F, et al. EGFR mutation status in Middle Eastern patients with non-squamous non-small cell lung carcinoma: A single institution experience. Cancer Epidemiol. 2015 Dec;39(6):1099-102. doi: 10.1016/j.canep.
5. Lai Y, Zhang Z, Li J, et al. EGFR mutations in surgically resected fresh specimens from 697 consecutive Chinese patients with non-small cell lung cancer and their relationships with clinical features. Int J Mol Sci. 2013 Dec 17;14(12):24549-59. doi: 10.3390/ijms141224549.
6. Zhang Z, Zhou S. Serum EGFR gene mutation status via second-generation sequencing and clinical features of patients with advanced lung cancer. Int J Clin Exp Pathol. 2018 Jul 1;11(7):3551-3558.
7. Wu JY, Shih JY. Effectiveness of tyrosine kinase inhibitors on uncommon E709X epidermal growth factor receptor mutations in non-small-cell lung cancer. Onco Targets Ther. 2016 Oct 11;9:6137-6145. doi: 10.2147/OTT.S118071.
8. Tu HY, Ke EE, Yang JJ, et al. A comprehensive review of uncommon EGFR mutations in patients with non-small cell lung cancer. Lung Cancer. 2017 Dec;114:96-102. doi: 10.1016/j.lungcan.2017.11.005.
9. Wei B, Ren P, Zhang C, et al. Characterization of common and rare mutations in EGFR and associated clinicopathological features in a large population of Chinese patients with lung cancer. Pathol Res Pract. 2017 Jul;213(7):749-758. doi: 10.1016/j.prp.2017.04.007. Epub 2017 Apr 20. Erratum in: Pathol Res Pract. 2017 Dec;213(12 ):1575.
10. Kobayashi Y, Togashi Y, Yatabe Y, et al. EGFR Exon 18 Mutations in Lung Cancer: Molecular Predictors of Augmented Sensitivity to Afatinib or Neratinib as Compared with First- or Third-Generation TKIs. Clin Cancer Res. 2015 Dec 1;21(23):5305-13. doi: 10.1158/1078-0432.CCR-15-1046.
11. John T, Akamatsu H, Delmonte A, et al. EGFR mutation analysis for prospective patient selection in AURA3 phase III trial of osimertinib versus platinum-pemetrexed in patients with EGFR T790M-positive advanced non-small-cell lung cancer. Lung Cancer. 2018 Dec;126:133-138. doi: 10.1016/j.lungcan.2018.10.027. Epub 2018 Nov 1.
